# Children with congenital heart disease exhibit seasonal variation in physical activity

**DOI:** 10.1371/journal.pone.0241187

**Published:** 2020-11-05

**Authors:** Mimi T. Y. Kuan, Christine Voss, Jimmy Lopez, Nicole M. Hemphill, Kevin C. Harris

**Affiliations:** 1 Children’s Heart Centre, BC Children’s Hospital, Vancouver, British Columbia, Canada; 2 Department of Pediatrics, University of British Columbia, Vancouver, British Columbia, Canada; University of Bern, University Hospital Bern, SWITZERLAND

## Abstract

**Objective:**

We sought to identify seasonal variation in physical activity that different physical activity measurement tools can capture in children with congenital heart disease.

**Methods:**

Data were collected as part of a prospective cohort study at BC Children’s Hospital, Vancouver, Canada. Daily step counts of children aged 9–16 years with moderate-to-severe CHD were assessed continuously for 1-year via a commercial activity tracker (Fitbit Charge 2^™^). Physical activity levels were also assessed conventionally at one time-point via accelerometers (ActiGraph) and physical activity questionnaires.

**Results:**

156 children (mean age 12.7±2.4 years; 42% female) participated in the study. Fitbit data (n = 96) over a 1-year period clearly illustrated seasonal peaks (late spring and autumn) and dips (winter and summer school holidays) in physical activity levels, with group mean values being below 12,000 steps per day throughout the year. According to conventional accelerometry data (n = 142), 26% met guidelines, which tended to differ according to season of measurement (spring: 39%, summer: 11%, fall: 20%, winter: 39%; p-value = 0.053). Questionnaire data (n = 134) identified that the most widely reported activities were walking (81%) and running (78%) with walking being the highest in summer and fall and running in winter and spring. Furthermore, regardless of overall activity levels the children exhibit similar seasonal variation.

**Conclusions:**

We demonstrated that physical activity level changes across seasons in children with CHD. It is important to be aware of these fluctuations when assessing and interpreting physical activity levels. Season specific counselling for physical activity may be beneficial in a clinical setting.

## Introduction

Congenital heart disease (CHD) is the most common congenital defect in new-borns occurring in approximately 1 in 100 live births [1]. Survival rates of children with moderate-to-complex forms of CHD have significantly increased and most of these individuals have a life expectancy that extends well into adulthood [2]. Consequently, the prevalence of CHD across all age ranges has increased [3, 4]. It is well established that the CHD populations are at an increased risk for cardiovascular events compared to the general population [5]. Physical activity is defined as “any bodily movement produced by skeletal muscle that results in energy expenditure” [6]; low physical activity is a modifiable risk factor and higher levels of physical activity are associated with better long-term cardiovascular risk in population-based studies [7]. Physical activity is an important determinant in optimizing the long-term cardiovascular health and quality of life in the CHD population [8].

Despite the beneficial effect of physical activity, levels of physical activity decline with age in children with CHD [9]. Adolescent declines in physical activity are particularly concerning given the increased cardiovascular risk of the CHD population [5]. Physical activity research in children with CHD is an emerging field of study; however, assessing physical activity can be challenging. Physical activity behaviours are highly heterogeneous between individuals, likely to vary over time, and may be impacted by their medical history [10] and other sociocultural factors [11, 12]. Seasonal variation of physical activity is the idea of how the fluctuation in weather, temperature, and daylight hours accompanying each season will impact levels of physical activity [13]. While seasonal variations have been extensively documented in the healthy pediatric population [13], there are no data on longitudinal physical activity patterns for children with CHD.

The aim of this study was to determine whether physical activity varies across seasons in children with CHD by measuring physical activity longitudinally (commercial activity trackers) and via conventional periodic measures (accelerometers and questionnaires).

## Materials and methods

### Sample

Data were collected as part of a prospective cohort study of children and adolescents aged 9–16 years old with Tetralogy of Fallot, Coarctation of the Aorta, Transposition of the Great Arteries, or Fontan circulation investigating the relationship between vascular function and physical activity and at the time of the current analyses, 156 children and youth had been enrolled in the study. Participants were recruited through the Children’s Heart Centre at BC Children’s Hospital in Vancouver, Canada, or at pediatric cardiology partnership clinics across British Columbia and the Yukon, Canada, between April 2017 and May 2019. Participants were excluded if they had health conditions that would prevent them from completing the study measures or participating in physical activity. Prior to study commencement, we obtained written informed parent/guardian consent and written informed participant assent. The study was approved by UBC Children’s & Women’s Research Ethics Board (H17-01233).

### Participant characteristics

We obtained participants sex, age, and cardiac diagnosis from patient medical charts. Trained clinical staff measured height (0.1cm) and weight (0.1kg). Body mass index (BMI; km/m^2^) was calculated and expressed as age- and sex-specific percentiles based on World Health Organization International growth charts [14]. BMI was categorized based on World Health Organization cut-offs. As part of a cohort study, clinical characteristics of this cohort have been described in detail [15].

### Physical activity measurements

#### Commercial activity tracker

We chose the Fitbit Charge 2^™^ (Fitbit Inc, San Francisco, CA) for our study. Wristband size and placement were in accordance with manufacturer’s guidelines. We asked participants to wear the Fitbit continuously for 12 months and sync the device on a regular basis. We created anonymous user profiles and used the ‘Data Export’ function from the online ‘dashboard’ to export daily step count data. In the absence of any consensus on wear time validation for commercial trackers in children, we considered a day to be valid if they had ≥1000 steps/d, and a month to be valid if they had ≥14 valid days. Monthly step counts were calculated by averaging the step counts from all the valid days of each month. We defined meeting physical activity guidelines as having on average ≥12,000 steps per day [16, 17].

#### Accelerometer

We fitted participants with an ActiGraph accelerometer (GT3X+, GT9X; ActiGraph LLC, Pensacola, FL) to be worn over the right hip for 7 days during waking hours and only to be removed for water-based activities. The ActiGraph is a commonly used tri-axial accelerometer to objectively measure physical activity levels in children under free-living condition [18–20]. We used ActiLife v.6.13.2 (ActiGraph LLC, Pensacola, FL) for accelerometer initialization (sampling set at 30Hz) and file download, processing, and analysis. We generated 15s epoch.agd files from the raw.gt3x files and used the wear time function in ActiLife to identify valid accelerometry files. We considered a day to be valid if the device was worn for ≥600 mins/day. We previously demonstrated that a minimum of 3 valid days are sufficient to estimate mean physical activity values in children and adolescents with CHD [9]. For valid accelerometry files, we used Evenson cut-points [21] to estimate mean daily minutes spent in moderate-to-vigorous physical activity (MVPA) intensity. We defined meeting physical activity guidelines as ≥60 minutes of MVPA per day, on average, and this is in accordance to the Canadian 24-Hour Movement Guidelines for Children and Youth [22]. We categorized participants’ physical activity levels based on the distribution of MVPA in our sample as follows: ‘low’ = <30 mins MVPA /day (~<25^th^ percentile); ‘medium’ = 30–59 mins MVPA/day (~25^th^–75^th^ percentile); ‘high’ = ≥60 mins MVPA/day (>75^th^ percentile).

#### Physical activity questionnaire (PAQ)

The Physical Activity Questionnaire for Children and the Physical Activity Questionnaire for Adolescents are appropriate for elementary school-age children and high school students respectively. The PAQ is a self-administered, 7-day recall questionnaire that assesses participation in different activities, as well as activity during Physical Education, lunch break, recess (PAQ-Children only), after school, in the evenings, and on weekends to provide general estimates of physical activity levels [23]. Participants completed the PAQ for Children (≤11yrs) or Adolescents (≥12yrs). We opted for this conservative age cut-off because secondary school usually starts with grade 8 and does not routinely offer recess. Both PAQ for Children and Adolescents demonstrated acceptable validity in measuring general levels of PA in children and adolescents [24, 25]. We previously demonstrated that the PAQ is valid for use in children with CHD [26]. The overall PAQ-score is derived from the mean scores of all questionnaire items. We identified the most common activities from the PAQ by calculating the proportion of children who self-reported to have participated in the activity during the previous 7 days. We identified the most commonly participated activities by season by counting the number of days the activity was performed in the past 7 days. We categorized participants’ activity levels based on the distribution of questionnaire scores in our sample as follows: ‘low’ = <2.7 PAQ-score (~<50^th^ percentile); ‘high’ = ≥2.7 PAQ-score (~≥50^th^ percentile).

### Procedures

We approached the participants during their routine clinical visit and provided the participants with a Fitbit Charge 2, an accelerometer and physical activity questionnaire. We asked participants to wear the Fitbit for the duration of the study and sync the device on a regular basis in order to assess physical activity longitudinally. Data from May 1, 2018 –April 30, 2019 were analyzed to evaluate seasonal variation. The participants wore the accelerometer over the right hip for 7 days and recorded the times in which the accelerometer was put on and removed. The questionnaire was self-administered in a quiet room during clinic visit or at home. Upon completion of the accelerometry, the participants mailed back the study material.

### Statistical analysis

Descriptive statistics (frequencies [%], mean±SD, or median [IQR]) were calculated for applicable variables. Distributions of continuous variables were assessed visually. The ‘lowess’ function in R was used to generate a physical activity line of best fit for the commercial activity tracker over a 1-year period. Between-season differences were assessed via one-way ANOVA (*post hoc* Bonferroni correction) for continuous variables or chi-square tests for categorical variables. All analyses were performed in R (version 3.6.0) using R Studio (version 1.1.442) and significance was set at *p* < 0.05. Seasons were defined as spring (March–May), summer (June–August), autumn (September–November), and winter (December–February).

## Results

### Sample

One hundred and fifty-six children were recruited to the study (mean age 12.7±2.4 years, 42% females) with at least one complete and valid commercial activity tracker, accelerometry, or physical activity questionnaire data. Overall participant characteristics are shown in Table 1. There is season-difference in the mean age for PAQ but not accelerometer. Other sample characteristics of accelerometer and PAQ are not significantly different between seasons (S1 and S2 Tables).

**Table 1:** Sample characteristics.

	All	Fitbit	Accelerometer	Physical Activity Questionnaire
N (%)	156 (100)	104 (66.7)	142 (91.0)	139 (89.1)
Female, n (%)	66 (42.3)	42 (40.4)	58 (40.8)	55 (40.0)
Age, years (mean, SD)	12.7 (2.4)	12.4 (2.3)	12.7 (2.4)	12.8 (2.4)
Height, cm (mean, SD)	152.2 (15.7)	151.2 (15.6)	152.2 (15.8)	152.5 (15.5)
Weight, kg (mean, SD)	46.5 (16.7)	46.1 (17.0)	47.0 (16.9)	46.2 (16.4)
BMI, percentile (mean, SD)	53.6 (33.4)	56.0 (34.0)	54.4 (33.5)	51.5 (33.4)
BMI Weight Category				
Thinness, n (%)	6 (3.9)	3 (2.9)	4 (2.8)	5 (3.6)
Normal, n (%)	111 (71.6)	71 (68.9)	101 (71.6)	101 (73.2)
Overweight, n (%)	21 (13.5)	16 (15.5)	19 (13.5)	18 (13.0)
Obese, n (%)	17 (11.0)	13 (12.6)	17 (12.1)	14 (10.1)
Cardiac Diagnosis				
COA, n (%)	47 (30.1)	31 (29.8)	42 (29.6)	43 (30.9)
TET, n (%)	38 (24.4)	24 (23.1)	33 (23.2)	33 (23.7)
TGA, n (%)	29 (18.6)	20 (19.2)	27 (19.0)	28 (20.1)
FON, n (%)	42 (26.9)	29 (27.9)	40 (28.2)	35 (25.2)

BMI—Body Mass Index (kg/m^2^); BMI percentiles calculation based on age-sex-specific World Health Organization 2007 reference charts [14].

BMI weight category based on World Health Organization cut-offs.

COA—Coarctation of the Aorta, TET—Tetralogy of Fallot, TGA—Transposition of the Great Arteries, FON—Fontan Circulation.

### Longitudinal physical activity patterns: Commercial activity trackers

**Fig 1:**
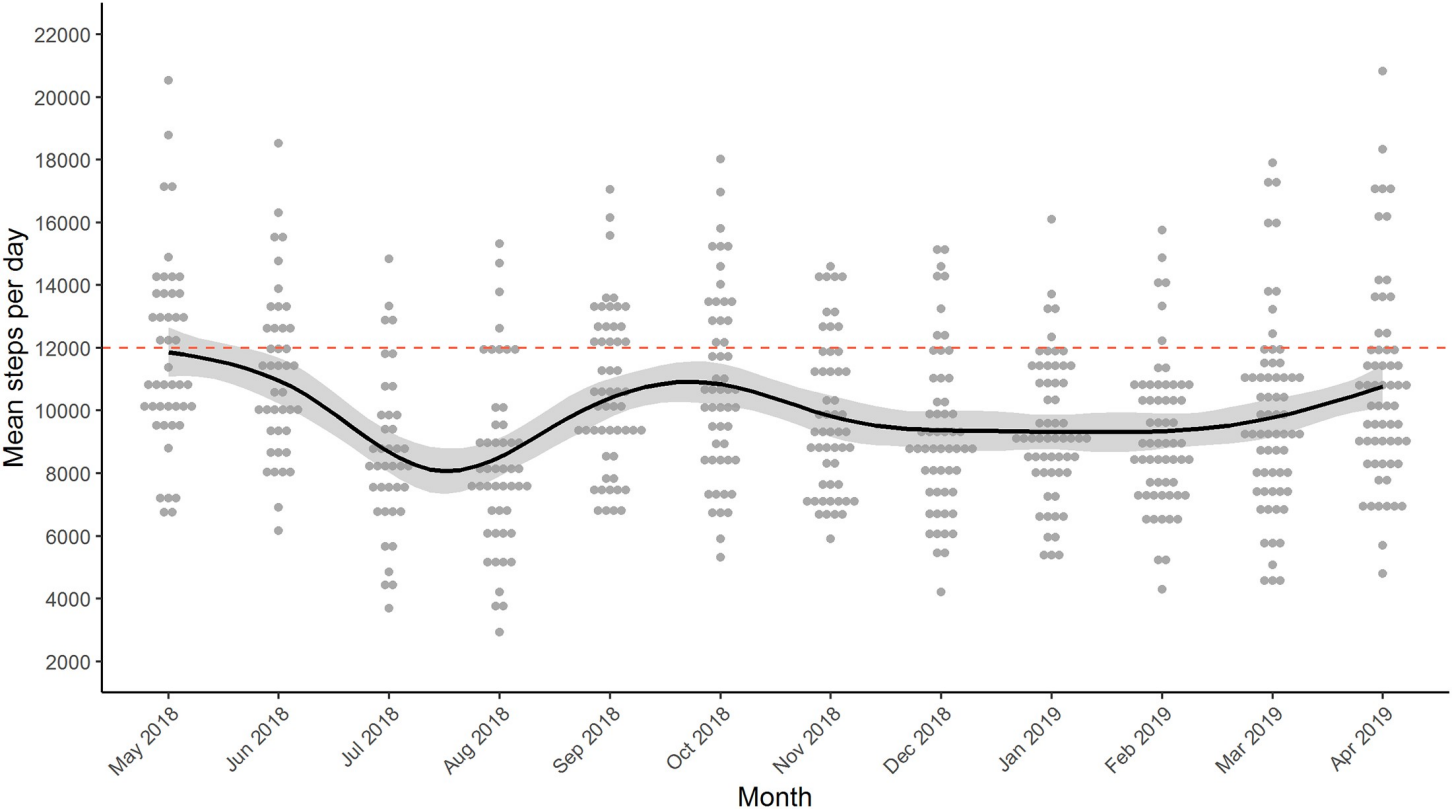
Seasonal variation from 1-year of Fitbit step count. LOWESS trend line illustrating seasonal variation over the course of a year with Fitbit step count data. The red dashed line is the target steps per day set for the participants’ Fitbit (12,000 steps). Data points in each month are shown side-by-side for visualization of clusters. Grey shaded area is the standard error.

Fitbit data were taken over a 1-year period from May 1, 2018 –April 30, 2019 (*n* = 96) and the physical activity line-of-best-fit is shown in Fig 1. This clearly illustrated seasonal peaks and dips in physical activity. The peaks occurred in late spring and autumn. There was a severe dip in July and August which corresponds to local school holidays. There was a long trough during the colder winter months. The mean step count was 9,960±2,874 steps per day and the group mean step count remained below 12,000 steps per day throughout the year (Table 2, n = 104). Season-differences in mean number of steps were lower during the summer and winter compared to spring and autumn (Table 2, *p<0*.*001)*. Overall, 23% of the children met the recommended step goal and 36%, 19%, 30%, and 12% of the children met the step goal in spring, summer, autumn, and winter respectively (Table 2, *p*<0.001).

**Table 2:** Physical activity characteristics by seasons.

	Overall	Spring	Summer	Autumn	Winter	P-value^Փ^
Commercial activity tracker						
N	104					
Steps (mean, SD)	9960 (2874)	10678 (3194)	9307 (2963)	10414 (2671)	9325 (2389)	0.000
Meet 12,000 steps, %^☐^		35.7	19.1	29.5	12.3	0.000
Accelerometer						
N	142	43	28	40	31	
MVPA, min/d (mean, SD)	45.5 (20.6)	48.9 (21.7)	39.3 (21.4)	43.5 (19.8)	48.7 (18.7)	0.183
Meet guidelines, n (%)^#^	37 (26.1)	14 (32.6)	3 (10.7)	8 (20.0)	12 (38.7)	0.053
Activity levels^¶^						
High, n (%)	37 (26.1)	14 (32.6)	3 (10.7)	8 (20.0)	12 (38.7)	0.045^†^
Medium, n (%)	71 (50.0)	18 (41.9)	14 (50.0)	26 (65.0)	13 (41.9)	
Low, n (%)	34 (23.9)	11 (25.6)	11 (39.3)	6 (15.0)	6 (19.4)	
Physical activity questionnaire						
N	139	47	28	34	30	
PAQ-score (mean, SD)^◊^	2.65 (0.71)	2.73 (0.76)	2.68 (0.71)	2.40 (0.65)	2.77 (0.66)	0.14
Activity levels^ǂ^						
High, n (%)	61 (43.9)	22 (46.8)	15 (53.6)	10 (29.4)	14 (46.7)	0.239
Low, n (%)	78 (56.1)	25 (53.2)	13 (46.4)	24 (70.6)	16 (53.3)	

MVPA—Moderate-to-Vigorous Physical Activity (min/day).

^Փ^ p-value for main effect for season.

^☐^ Proposed daily step target to determine if children are meeting physical activity guideline [16].

^#^ Meeting physical activity guidelines is defined as mean daily minutes of moderate-to-vigorous physical activity per day of ≥60 minutes.

^¶^ Accelerometry-derived activity level from our cohort: high ((≥60 mins/day), medium (30–50 mins/day), low (<30 mins/day).

^†^ No significant Bonferroni-adjusted *post hoc* comparisons present between groups (*p<*0.008).

^◊^ Scored on a scale from 1 (no/low activity) to 5 (very active) [23].

^ǂ^ PAQ-score-derived activity level from our cohort: high (≥2.7), low (<2.7).

### Periodic physical activity levels: Accelerometry

The mean moderate-to-vigorous physical activity (MVPA) was 45.5±20.6 minutes per day (Table 2, n = 142). There were no significant between season-differences (*p* = 0.183). Overall, 26% of the children with CHD met physical activity guidelines and 33%, 11%, 20%, and 39% met guidelines in the spring, summer, autumn, and winter respectively.

### Periodic physical activity levels: Physical activity questionnaires

The mean PAQ-score was 2.7±0.7 (Table 2, n = 139). There were no significant between season-differences in PAQ-score (*p* = 0.14) or PAQ-score derived activity levels (*p* = 0.239). Proportion of physical activities participation can be found in Table 3. The five most commonly reported activities are walking (81%), running (78%), tag (48%), basketball (40%), and bicycling (39%). Table 4 depicts the six most common activities by season in percentage of days. While walking and running were found to be the most common activities throughout the year, other structured and unstructured activities demonstrated seasonal variation. Unstructured activity (eg. tag) decreased in frequency when children are on school holidays. Structured activities such as basketball and soccer were more prevalent during the school year.

**Table 3:** Physical activities frequency table.

	Proportion of children who participated
Physical Activiy	
Walking	80.6%
Running	77.7%
Tag	47.5%
Basketball	40.3%
Bicycling	38.8%
Soccer	36.0%
Dance	33.8%
Swimming	30.2%
Skipping	18.7%
Volleyball	17.3%
Badminton	15.1%
Baseball	12.9%
Street Hockey	12.9%
Aerobics	11.5%
Football	10.1%
Floor Hockey	9.4%
Ice Skating	7.9%
Skateboarding	7.2%
Ice Hockey	6.5%
In-line Skating	2.9%

**Table 4:** Ranking in percentage days of top 6 activities participation by season.

Spring		Summer		Autumn		Winter	
Activity	% Days	Activity	% Days	Activity	% Days	Activity	% Days
Running	47.4%	Walking	53.1%	Walking	51.7%	Running	46.4%
Walking	44.7%	Running	30.4%	Running	34.5%	Walking	31.7%
Tag	25.7%	Bicycling	27.0%	Soccer	18.1%	Tag	20.0%
Bicycling	23.3%	Swimming	21.7%	Tag	17.4%	Basketball	19.0%
Basketball	18.4%	Dance	18.0%	Bicycling	15.3%	Bicycling	15.0%
Dance	15.0%	Tag	13.5%	Dance	13.4%	Soccer	15.0%

### Seasonal variation in step count stratified by physical activity levels using conventional measures

Plotting of participants’ longitudinal Fitbit data stratified by their accelerometry-derived MVPA activity level (high, medium, and low) produced distinct fitted lines for these three groups across seasons (S1 Fig). The standard errors of the three best fit lines showed minimal overlap amongst the groups. The three groups followed similar physical activity patterns across seasons (i.e. physical activity levels were highest in late spring). There was a significant association between accelerometry-derived MVPA minutes per day and Fitbit step counts (*r* = 0.73; *p* = 0.000; R-squared: 0.54). However, Fitbits overestimate step counts compared to accelerometers with a mean bias of 1694 steps (95% confidence interval: 590–2798 steps; *p* = 0.003).

Plotting of participants’ longitudinal Fitbit data stratified by their PAQ-score derived activity level (high and low) produced distinct fitted lines for these two groups across seasons (S2 Fig). The standard errors of the two best fit lines showed minimal overlap amongst the groups. The two groups followed similar physical activity patterns across seasons (i.e. physical activity levels were highest in late spring). Associations between PAQ-scores and Fitbit step counts were significant (*r* = 0.355; *p* = 0.013; R-squared: 0.13).

## Discussion

Our study demonstrated that continuous measurements of physical activity with commercial activity trackers clearly capture seasonal variation in physical activity in children with CHD. A key strength of our study is the use of both longitudinal (commercial activity trackers) and periodic (accelerometers and questionnaires) measurement tools to quantify physical activity levels of children with CHD throughout the year.

### Seasonal variation

We demonstrated that commercial activity trackers, which are able to provide continuous measurement of physical activity level in the form of step count, are able to capture seasonal variation over a 1-year period. In contrast with other studies [13, 27] that made assumptions regarding an increase in activity level throughout the summer months due to warm weather and long daylight hours, our use of a continuous monitor clearly showed that activity levels dip in July and August. This is an important observation given that this drop corresponds directly with local school holidays. This fluctuation that other studies weren’t able to capture was likely due to the widely used measurement tools of accelerometer and questionnaire where data are typically collected periodically during the school year [13].

Conventional methods, such as accelerometer, are commonly used to objectively measure physical activity in population-based studies (NHANES [18], CHMS [19]). In our study, 26% of children with CHD achieved a weekly average of at least 60 minutes per day compared to the Canadian national average of 33% [19]. Seasonal variation of the accelerometry data demonstrated that only 11% of the children were able to meet guidelines during the summer period, which is notably lower than the proportion of children meeting guidelines during the other seasons (20–40%). This demonstrates that for researchers who use accelerometers to measure physical activity levels in children, there is a possibility of miscategorizing children with respect to physical activity guidelines. Seasonal influences on physical activity may also impact the results seen in short-term physical activity intervention programs if pre- and post-intervention measurements are taken at different times of the year.

In our experience, the PAQ has limited capability in discerning activity levels in children with CHD because children tend to be clustered in a narrow range on the 5-point scale (IQR: 2.2–3.1). However, the PAQ provides useful insight on the types of activities children participate in and how those vary across seasons. We demonstrated that certain activities are more prevalent during different times of the year. For example, cycling is more common in the summer, whereas, basketball is more common in the winter. Therefore, it is important for healthcare providers to recognize this since the promotion of season-specific activities at the correct time of the year may be beneficial in increasing activity levels.

Our data suggest that physical activity participation in children with CHD is impacted by the school-year. There are two mechanisms by which school is known to facilitate physical activity [28], and these likely impact children with CHD as they do typical children. When school is in session, there is significant school-associated physical activity (i.e. active transportation, play before and after school and during recess and lunch on the school grounds, and physical education) and participation in sport programs orientated around the school calendar (i.e. school or community-based sports teams or clubs) [28]. Our data also imply that weather acts as a barrier for physical activity participation [29]. Children’s activity levels are blunted during the winter months due to colder temperature and shorter daylight hours despite being in school and getting the physical activity benefits associated with the school year.

Conventional measurement tools and commercial activity trackers provide different and complementary insight into physical activity patterns. With our combined dataset, we demonstrated that seasonal patterns hold for all children regardless of their baseline activity levels, with activity peaking in late spring and dropping during summer holidays and the winter months. This underscores the importance of repeated physical activity promotion for all children with CHD, especially during the months when children are known to be less active (summer and winter). Recognizing that these patterns are pervasive amongst children may help clinicians and exercise physiologists counsel children with season-specific advice. We also demonstrated that commercial activity trackers can portray long-term activity patterns and may be the more appropriate tool to use when studying seasonal variation or conducting long-term monitoring of physical activity.

### Clinical implications

In our longitudinal analysis, we demonstrated that levels of physical activity vary by season in children with CHD. Therefore, it is important for physicians to discuss these fluctuations with families while promoting physical activity participation. Our results suggest that the promotion of physical activity types should be targeted based on the time of the year. Furthermore, discussing the natural fluctuations in physical activity levels may help families identify opportunities to increase physical activity levels at observed nadirs. Summer holidays are a particularly good opportunity for family-based activity promotion.

Simple tools like the PAQ can easily be administered in a clinical setting and be used to identify activities with high level of participation throughout the year. Such information can be used to help identify targets for promotion and facilitation of physical activity by their primary caretakers (i.e. during the summer time, can you try cycling for 10 minutes every day?).

Given that all children demonstrate seasonal variation regardless of baseline activity levels, the incorporation of information from commercial activity tracker, accelerometer, and PAQ provides a comprehensive picture to optimise season-specific physical activity guidance.

### Research implications

Recognizing and understanding the effect of seasonal variation is important for researchers who use periodic measurement tools (accelerometer and questionnaires). Our results demonstrate that caution is needed when comparing cross-sectional results using these conventional physical activity measurement tools. Our study shows the feasibility of monitoring physical activity in children over the course of an entire year. These data provide depth compared to cross-sectional measurements, which clearly have important limitations given the seasonal variation we have shown. Commercial activity trackers can play an important role in interventional studies, as compliance with the intervention can be measured continuously before, during, and after the intervention as opposed to the usual approach of before and after measurements with conventional tools.

### Strengths and limitations

To our knowledge, this is the first study to use commercial activity trackers in children with CHD to assess activity patterns over a 1-year period. There are important limitations to physical activity trackers which must be noted. In agreement with our previous Fitbit validation study in children with CHD [17], the Fitbit device overestimated step counts in the current study compared to the accelerometer.

A limitation of accelerometers is low adherence (~65%); however, in our cohort, ~90% of the children adhered to wear-time protocol and provided sufficient data compared to other studies [19, 30]. In addition, the Fitbit was well-received with good initial adherence to protocols. Over the 1-year period, data capture rate dropped to ~60% due to loss of interest, technical difficulties, or skin irritations (rashes, eczema). Nevertheless, the adherence for 1-year of continuous data capture remains very good and was positively impacted by reminders from the study team to the children’s guardians. For the PAQ, it is important to note that it is subjected to recall bias and children tend to over-estimate activity levels [31]. However, in our cohort, PAQ-scores were significantly correlated with Fitbit and accelerometry data.

Although season-differences were not significant with accelerometry-derived MVPA or PAQ-score, the seasonal effects may have been masked by the small number of participants in each season and the wide distribution of physical activity levels amongst children. An additional limitation of the PAQ—one of the few suitable questionnaire tools available for use in children and youths [32]—is that it does not provide valid estimates of physical activity outside of the school year [23] and, therefore, would be of limited accuracy in detecting these seasonal changes. Commercial activity trackers overcome these limitations and are well liked by children resulting in high rates of wear time compliance.

While we have a large sample size of 156 children in the study, they came from a geographically diverse area where temperature and precipitation within a season varied. However, subgroup analysis was not performed due to insufficient power in each geographic region. Such analysis would also not be meaningful as it does not account for seasonal participation in physical activity, daylight hours [33], and other geographic factors (rural vs. urban) [34].

## Conclusions

Recognizing the effect of seasonality in physical activity in children with CHD may help healthcare providers administer effective physical activity counselling at each annual routine care encounter. Our data demonstrated that commercial activity trackers are a powerful tool to capture, assess, and understand variations in activity levels over long periods of time. Researchers should consider the significant confounding role of seasonality on physical activity behaviour in the design, implementation, and evaluation of interventions.

## Supporting information

S1 Fig Seasonal variation stratified by accelerometry. Distinct LOWESS trend lines of children’s longitudinal Fitbit data categorized by their activity levels from their accelerometry data. High activity level (green) are children who achieved an average of ≥60 minutes of moderate-to-vigorous physical activity per day (MVPA) from their accelerometry data. Medium activity level (blue) are children who achieved an average of 30–59 minutes of MVPA per day. Low activity level (purple) are children who achieved an average of <30 minutes of MVPA per day. Grey area is the standard error.
(TIFF)

S2 Fig Seasonal variation stratified by physical activity questionnaire. Distinct LOWESS trend lines of children’s longitudinal Fitbit data categorized by their activity levels from their PAQ-scores. High activity level (green) are children who achieved ≥2.7 PAQ-score from their questionnaire response. Low activity level (purple) are children who achieved <2.7 PAQ-score. Grey area is the standard error.
(TIFF)

S1 Table Sample characteristic of accelerometer by seasons. (PDF)

S2 Table Sample characteristic of physical activity questionnaire by seasons. (PDF)

## References

[1] van der Linde D, Konings EE, Slager MA, Witsenburg M, Helbing WA, Takkenberg JJ, et al Birth prevalence of congenital heart disease worldwide: A systematic review and meta-analysis. J Am Coll Cardiol. 2011;58(21):2241–7. 10.1016/j.jacc.2011.08.025 22078432

[2] Marelli AJ, Mackie AS, Ionescu-Ittu R, Rahme E, Pilote L. Congenital heart disease in the general population. Circulation. 2007;115(2):163–72. 10.1161/CIRCULATIONAHA.106.627224 17210844

[3] Marelli AJ, Ionescu-Ittu R, Mackie AS, Guo L, Dendukuri N, Kaouache M. Lifetime prevalence of congenital heart disease in the general population from 2000 to 2010. Circulation. 2014;130(9):749–56. 10.1161/CIRCULATIONAHA.113.008396 24944314

[4] Khairy P, Ionescu-Ittu R, Mackie AS, Abrahamowicz M, Pilote L, Marelli AJ. Changing mortality in congenital heart disease. J Am Coll Cardiol. 2010;56(14):1149–57. 10.1016/j.jacc.2010.03.085 20863956

[5] Bouma Berto J, Mulder Barbara JM. Changing landscape of congenital heart disease. Circ Res. 2017;120(6):908–22. 10.1161/CIRCRESAHA.116.309302 28302739

[6] Caspersen CJ, Powell KE, Christenson GM. Physical activity, exercise, and physical fitness: Definitions and distinctions for health-related research. Public Health Rep. 1985;100(2):126–31. 3920711 PMC1424733

[7] Pälve Kristiina S, Pahkala K, Magnussen Costan G, Koivistoinen T, Juonala M, Kähönen M, et al Association of physical activity in childhood and early adulthood with carotid artery elasticity 21 years later: The cardiovascular risk in young finns study. J Am Heart Assoc.3(2):e000594 10.1161/JAHA.113.000594 24755150 PMC4187482

[8] Longmuir PE, Brothers JA, de Ferranti SD, Hayman LL, Van Hare GF, Matherne GP, et al Promotion of physical activity for children and adults with congenital heart disease: A scientific statement from the american heart association. Circulation. 2013;127(21):2147–59. 10.1161/CIR.0b013e318293688f 23630128

[9] Voss C, Duncombe SL, Dean PH, de Souza AM, Harris KC. Physical activity and sedentary behavior in children with congenital heart disease. J Am Heart Assoc. 2017;6(3). 10.1161/JAHA.116.004665 28264859 PMC5524004

[10] Banks L, Rosenthal S, Longmuir PE, Manlhiot C, McKillop A, McCrindle BW. Medical factors associated with moderate-to-vigorous physical activity in children with congenital heart disease may be specific to the underlying lesion. Can J Cardiol. 2014;30(10, Supplement):S188–S9. 10.1007/s00246-017-1645-2 28608149

[11] Dollman J. Social and environmental influences on physical activity behaviours. Int J Environ Res Public Health. 2018;15(1):169 10.3390/ijerph15010169 29361761 PMC5800268

[12] Wilk P, Clark AF, Maltby A, Smith C, Tucker P, Gilliland JA. Examining individual, interpersonal, and environmental influences on children’s physical activity levels. SSM Popul Health. 2018;4:76–85. 10.1016/j.ssmph.2017.11.004 29349276 PMC5769121

[13] Carson V, Spence JC. Seasonal variation in physical activity among children and adolescents: A review. Pediatr Exerc Sci. 2010;22(1):81–92. 10.1123/pes.22.1.81 20332542

[14] de Onis M, Onyango AW, Borghi E, Siyam A, Nishida C, Siekmann J. Development of a who growth reference for school-aged children and adolescents. Bull World Health Organ. 2007;85(9):660–7. 10.2471/blt.07.043497 18026621 PMC2636412

[15] Lopez JR, Voss C, Kuan MTY, Hemphill NM, Sandor GGS, Harris KC. Physical activity is associated with better vascular function in children and adolescents with congenital heart disease. Can J Cardiol. 2020;36(9):1474–81. 10.1016/j.cjca.2019.12.019 32603699

[16] Colley RC, Janssen I, Tremblay MS. Daily step target to measure adherence to physical activity guidelines in children. Med Sci Sports Exerc. 2012;44(5):977–82. 10.1249/MSS.0b013e31823f23b1 22051570

[17] Voss C, Gardner RF, Dean PH, Harris KC. Validity of commercial activity trackers in children with congenital heart disease. Can J Cardiol. 2017;33(6):799–805. 10.1016/j.cjca.2016.11.024 28347581

[18] Troiano RP, Berrigan D, Dodd KW, Masse LC, Tilert T, McDowell M. Physical activity in the united states measured by accelerometer. Med Sci Sports Exerc. 2008;40(1):181–8. 10.1249/mss.0b013e31815a51b3 18091006

[19] Colley RC, Carson V, Garriguet D, Janssen I, Roberts KC, Tremblay MS. Physical activity of canadian children and youth, 2007 to 2015. Health reports. 2017;28(10):8–16. 29044441

[20] Sherar LB, Griew P, Esliger DW, Cooper AR, Ekelund U, Judge K, et al International children’s accelerometry database (icad): Design and methods. BMC Public Health. 2011;11:485 10.1186/1471-2458-11-485 21693008 PMC3146860

[21] Evenson KR, Catellier DJ, Gill K, Ondrak KS, McMurray RG. Calibration of two objective measures of physical activity for children. J Sports Sci. 2008;26(14):1557–65. 10.1080/02640410802334196 18949660

[22] Tremblay MS, Carson V, Chaput J-P, Connor Gorber S, Dinh T, Duggan M, et al Canadian 24-hour movement guidelines for children and youth: An integration of physical activity, sedentary behaviour, and sleep. Appl Physiol Nutr Metab. 2016;41(6 (Suppl. 3)):S311–S27. 27306437 10.1139/apnm-2016-0151

[23] Kowalski KC, Crocker PR, Donen RM. The physical activity questionnaire for older children (paq-c) and adolescents (paq-a) manual. College of Kinesiology, University of Saskatchewan. 2004;87(1):1–38.

[24] Kowalski KC, Crocker PRE, Faulkner RA. Validation of the physical activity questionnaire for older children. Pediatr Exerc Sci. 1997;9(2):174.

[25] Kowalski KC, Crocker PRE, Kowalski NP. Convergent validity of the physical activity questionnaire for adolescents. Pediatr Exerc Sci. 1997;9(4):342.

[26] Voss C, Dean PH, Gardner RF, Duncombe SL, Harris KC. Validity and reliability of the physical activity questionnaire for children (paq-c) and adolescents (paq-a) in individuals with congenital heart disease. PLoS One. 2017;12(4):e0175806 10.1371/journal.pone.0175806 28445485 PMC5406026

[27] Bélanger M, Gray-Donald K, O’Loughlin J, Paradis G, Hanley J. Influence of weather conditions and season on physical activity in adolescents. Ann Epidemiol. 2009;19(3):180–6. 10.1016/j.annepidem.2008.12.008 19217000

[28] Institute of Medicine (U.S.). Committee on Physical Activity and Physical Education., Kohl HW, Cook HD, National Academies Press Free eBooks Educating the student body: Taking physical activity and physical education to school [text] 2013 Click here for full text http://GW2JH3XR2C.search.serialssolutions.com/?sid=sersol&SS_jc=TC0001214173&title=Educating%20the%20student%20body%20%3A%20taking%20physical%20activity%20and%20physical%20education%20to%20school. 24851299

[29] Voss C, Duncombe S, Dean P, Harris K. Wearable activity trackers provide valuable insight into seasonal and school-related physical activity patterns in children with congenital heart disease. Can J Cardiol. 2017;33(10). 10.1016/j.cjca.2016.11.024 28347581

[30] Fairclough S, Noonan R, Rowlands A, van Hees V, Knowles Z, Boddy L. Wear compliance and activity in children wearing wrist and hip-mounted accelerometers. Med Sci Sports Exerc. 2015;48(2):245–53. 10.1249/MSS.0000000000000771 26375253

[31] Welk GJ, Corbin CB, Dale D. Measurement issues in the assessment of physical activity in children. Res Q Exerc Sport. 2000;71(sup2):59–73. 25680015 10.1080/02701367.2000.11082788

[32] Biddle SJH, Gorely T, Pearson N, Bull FC. An assessment of self-reported physical activity instruments in young people for population surveillance: Project alpha. Int J Behav Nutr Phys Act. 2011;8:1-. 10.1186/1479-5868-8-1 21194492 PMC3019119

[33] Goodman A, Paskins J, Mackett R. Day length and weather effects on children’s physical activity and participation in play, sports, and active travel. J Phys Act Health. 2012;9(8):1105–16. 10.1123/jpah.9.8.1105 22826506 PMC3584676

[34] Nadeau C, Letarte L, Fratu R, Waygood EOD, Lebel A. Does where you live matter? Leisure-time physical activity among canadian youth: A multiple cross-sectional study. CMAJ Open. 2016;4(3):E436–E43. 10.9778/cmajo.20150089 27730107 PMC5047838

