# Supplementary figures and images for: Children with congenital heart disease exhibit seasonal variation in physical activity
Source: PLoS One. 2020 Nov 5;15(11):e0241187. doi: 10.1371/journal.pone.0241187 (PMC7644044; doi:10.1371/journal.pone.0241187)

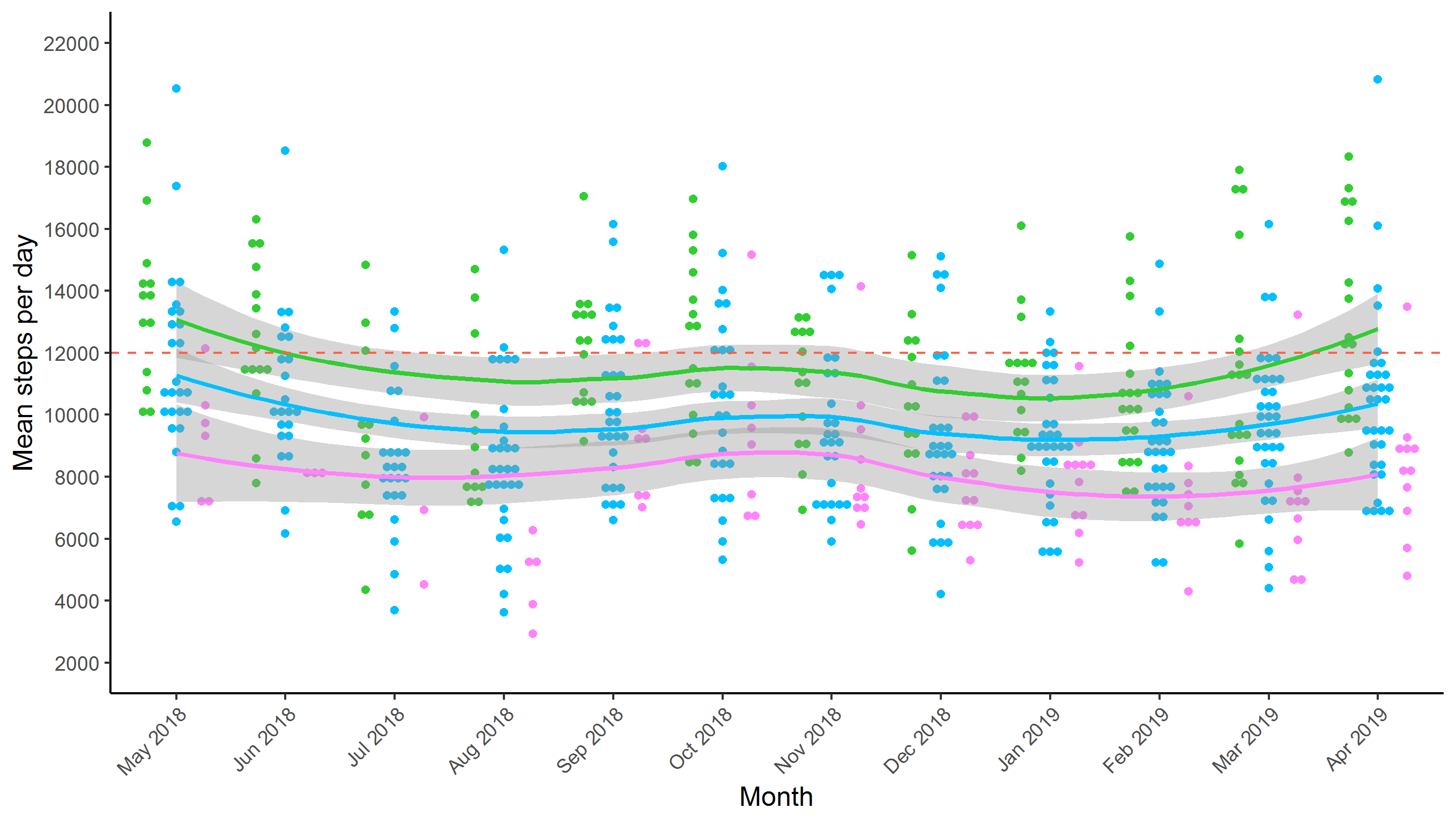

Supplement: S1 Fig — Distinct LOWESS trend lines of children’s longitudinal Fitbit data categorized by their activity levels from their accelerometry data. High activity level (green) are children who achieved an average of ≥60 minutes of moderate-to-vigorous physical activity per day (MVPA) from their accelerometry data. Medium activity level (blue) are children who achieved an average of 30–59 minutes of MVPA per day. Low activity level (purple) are children who achieved an average of <30 minutes of MVPA per day. Grey area is the standard error. (TIFF) [file pone.0241187.s001.tiff]

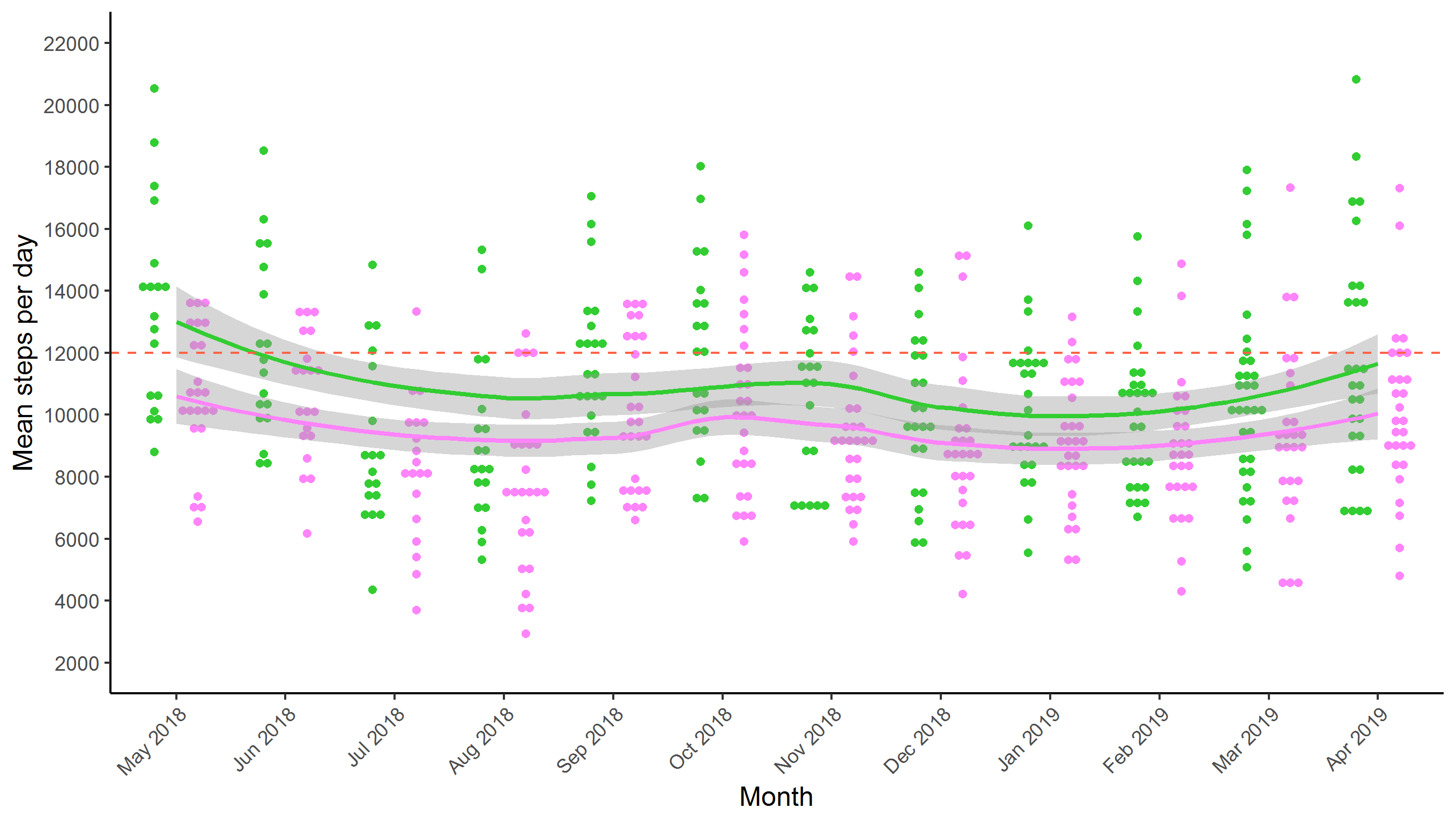

Supplement: S2 Fig — Distinct LOWESS trend lines of children’s longitudinal Fitbit data categorized by their activity levels from their PAQ-scores. High activity level (green) are children who achieved ≥2.7 PAQ-score from their questionnaire response. Low activity level (purple) are children who achieved <2.7 PAQ-score. Grey area is the standard error. (TIFF) [file pone.0241187.s002.tiff]
