# Supplementary material for: Children with congenital heart disease exhibit seasonal variation in physical activity
Source: PLoS One. 2020 Nov 5;15(11):e0241187. doi: 10.1371/journal.pone.0241187 (PMC7644044; doi:10.1371/journal.pone.0241187)
Supplement: S1 Table — (PDF) [file pone.0241187.s003.pdf]

**S1 Table. Sample characteristics of accelerometer by seasons**

| | Spring | Summer | Autumn | Winter | P-value$^{\Phi}$ |
|---|---|---|---|---|---|
| Accelerometer | | | | | |
| N | 43 | 28 | 40 | 31 | 0.230 |
| Female, n (%) | 18 (41.9) | 12 (42.9) | 14 (35.0) | 14 (45.1) | 0.833 |
| Age, years (mean, SD) | 12.3 (2.2) | 13.7 (2.3) | 13.0 (2.4) | 12.0 (2.5) | 0.022$^{\dagger}$ |
| Height, cm (mean, SD) | 151.2 (16.4) | 157.8 (16.2) | 154.1 (15.5) | 147.6 (13.5) | 0.078 |
| Weight, kg (mean, SD) | 46.7 (16.4) | 50.0 (17.0) | 48.7 (18.4) | 42.4 (15.3) | 0.307 |
| BMI, percentile (mean, SD) | 57.9 (35.5) | 48.1 (34.2) | 55.5 (32.4) | 53.9 (32.2) | 0.684 |
| BMI Weight Category | | | | | |
| Thinness, n (%) | 0 (0.0) | 2 (7.1) | 1 (2.5) | 1 (3.2) | |
| Normal, n (%) | 27 (64.3) | 21 (75.0) | 31 (77.5) | 22 (71.0) | 0.431 |
| Overweight, n (%) | 6 (14.3) | 4 (14.3) | 4 (10.0) | 5 (16.1) | |
| Obese, n (%) | 9 (21.4) | 1 (3.6) | 4 (10.0) | 3 (9.7) | |
| Cardiac Diagnosis | | | | | |
| COA, n (%) | 17 (39.5) | 6 (21.4) | 10 (25.0) | 9 (29.0) | |
| TET, n (%) | 9 (20.9) | 8 (28.6) | 10 (25.0) | 6 (19.4) | 0.858 |
| TGA, n (%) | 7 (16.3) | 7 (25.0) | 7 (17.5) | 6 (19.4) | |
| FON, n (%) | 10 (23.3) | 7 (25.0) | 13 (32.5) | 10 (32.2) | |

BMI – Body Mass Index ($kg/m^2$); BMI percentiles calculation based on age-sex-specific World Health Organization 2007 reference charts[14]
BMI weight category based on World Health Organization cut-offs
COA – Coarctation of the Aorta, TET – Tetralogy of Fallot, TGA – Transposition of the Great Arteries, FON – Fontan Circulation
$^{\Phi}$ p-value for main effect for season
$^{\dagger}$ No significant Bonferroni-adjusted *post hoc* comparisons present between groups ($p$<0.008)
