# Supplementary material for: Children with congenital heart disease exhibit seasonal variation in physical activity
Source: PLoS One. 2020 Nov 5;15(11):e0241187. doi: 10.1371/journal.pone.0241187 (PMC7644044; doi:10.1371/journal.pone.0241187)
Supplement: S2 Table — (PDF) [file pone.0241187.s004.pdf]

S2 Table. Sample characteristics of physical activity questionnaire by seasons

	Spring	Summer	Autumn	Winter	P-value$^{\Phi}$
Physical activity questionnaire					
N	47	28	34	30	0.098
Female, n (%)	16 (34.0)	15 (53.6)	13 (38.2)	11 (36.7)	0.388
Age, years (mean, SD)	12.3 (2.1)	13.8 (2.6)	13.4 (2.3)	11.8 (2.2)	0.003
Height, cm (mean, SD)	150.9 (15.5)	157.1 (17.6)	155.8 (14.4)	147.0 (13.3)	0.040†
Weight, kg (mean, SD)	44.8 (14.5)	51.6 (19.5)	49.0 (18)	40.4 (12.2)	0.043†
BMI, percentile (mean, SD)	53.1 (35.8)	51.3 (35.1)	49.5 (33.6)	51.4 (29.1)	0.973
BMI Weight Category					
Thinness, n (%)	2 (4.3)	1 (3.6)	1 (2.9)	1 (3.3)	
Normal, n (%)	30 (65.2)	20 (71.4)	27 (79.4)	24 (80.0)	0.955
Overweight, n (%)	7 (15.2)	4 (14.3)	4 (11.8)	3 (10.0)	
Obese, n (%)	7 (15.2)	3 (10.7)	2 (5.9)	2 (6.7)	
Cardiac Diagnosis					
COA, n (%)	18 (38.3)	7 (25.0)	9 (26.5)	9 (30.0)	
TET, n (%)	11 (23.4)	10 (35.7)	7 (20.6)	5 (16.7)	0.730
TGA, n (%)	8 (17.0)	6 (21.4)	7 (20.6)	7 (23.3)	
FON, n (%)	10 (21.3)	5 (17.9)	11 (32.4)	9 (30.0)	

BMI – Body Mass Index (kg/m^2); BMI percentiles calculation based on age-sex-specific World Health Organization 2007 reference charts[14]
BMI weight category based on World Health Organization cut-offs
COA – Coarctation of the Aorta, TET – Tetralogy of Fallot, TGA – Transposition of the Great Arteries, FON – Fontan Circulation
$^{\Phi}$ p-value for main effect for season
† No significant Bonferroni-adjusted *post hoc* comparisons present between groups ($p<0.008$)
